# Prospective, randomized, controlled trial comparing PROpofol versus KetaMINE in rapid sequence intubation in critically ill patients (PROMINE): protocol paper and statistical analysis plan

**DOI:** 10.62675/2965-2774.20250133

**Published:** 2025-11-03

**Authors:** Raysa Cristina Schmidt, Fernando Godinho Zampieri, Fernando Jose da Silva Ramos, Felipe Santos Cavatoni Serra, Lucas Petri Damiani, Flávio Geraldo Rezende de Freitas, Flávia Ribeiro Machado

**Affiliations:** 1 Universidade Federal de São Paulo Escola Paulista de Medicina Hospital São Paulo São Paulo SP Brazil Intensive Care Department, Hospital São Paulo, Escola Paulista de Medicina, Universidade Federal de São Paulo - São Paulo (SP), Brazil.; 2 Universidade Federal de São Paulo Escola Paulista de Medicina São Paulo SP Brazil Postgraduate Program in Translational Medicine, Escola Paulista de Medicina, Universidade Federal de São Paulo - São Paulo (SP), Brazil.; 3 Hospital do Serviço Social da Indústria do Papel, Papelão e Cortiça do Estado de São Paulo São Paulo SP Brazil Hospital do Serviço Social da Indústria do Papel, Papelão e Cortiça do Estado de São Paulo - São Paulo (SP), Brazil.

**Keywords:** Rapid sequence induction and intubation, Intubation, Airway management, Propofol, Ketamine, Hypnotics and sedatives

## Abstract

**Background:**

The optimal and safest hypnotic agent for rapid sequence intubation in critically ill patients remains uncertain. Factors such as hypovolemia, vasoplegia, hypoxemia, and acidosis can influence the efficacy and safety of induction agents. Propofol is commonly used for this purpose; however, it is associated with the risk of exacerbating hypotension. Ketamine, which has a more favorable hemodynamic profile, may offer a safer alternative in these patients.

**Objective:**

To assess whether ketamine is a safer alternative to propofol for rapid sequence intubation by reducing the incidence of hypotension during induction in critically ill patients.

**Methods:**

This will be a randomized, open-label, pragmatic, bicenter study. A total of 170 critically ill patients requiring endotracheal intubation in the intensive care unit will be randomly assigned to receive either ketamine or propofol as the hypnotic agent. Randomization will be conducted using RedCap with a 1:1 ratio and variable block sizes, stratified by study site and vasopressor use during intubation.

**Results:**

The primary outcome will be the occurrence of hypotension, defined as the lowest mean arterial pressure recorded within the first 10 minutes following induction. Secondary outcomes, assessed within 1-hour post-induction, include mortality, incidence of cardiopulmonary arrest, the occurrence of severe hypotension (systolic blood pressure < 80mmHg), the occurrence of severe hypoxemia (oxygen saturation < 85%), and the number of intubation attempts.

**Conclusion:**

The PROMINE study will provide valuable evidence to guide the selection of hypnotic agents for rapid sequence intubation in critically ill patients. It will contribute to a better understanding of the hemodynamic effects associated with propofol and ketamine in this context, potentially informing clinical practice.

## INTRODUCTION

Rapid sequence intubation (RSI) is a series of actions aimed at promptly and safely securing a definitive airway via orotracheal intubation in patients at risk of aspiration,^(1)^ However, this procedure may result in hemodynamic disturbances.^(2,3)^

The RSI algorithm typically involves pre-medication, induction with a hypnotic agent, and neuromuscular blockade before laryngoscopy.^(4)^ The primary hypnotic agents utilized for this purpose include propofol and ketamine.^(5)^ Propofol, an alkylphenol derivative, is a highly lipophilic agent with potent hypnotic properties. However, it may negatively affect myocardial contractility and vasodilation, resulting in hypotension.^(4)^ In contrast, ketamine, a phencyclidine derivative, exhibits cardiovascular stimulant effects that may increase blood pressure, heart rate (HR), and cardiac output, leading to an increase in myocardial oxygen demand and a potential rise in intracranial pressure.^(6)^ However, in critically ill patients, physiological alterations induced by stress and disease processes may modulate the hemodynamic response to ketamine, and some studies have also reported hypotension associated with its use.^(7,8)^

Several studies have compared various RSI agents and their effect on hemodynamics,^(4)^ though most of these studies were conducted in operating rooms^(9)^ or emergency departments.^(10,11)^ Despite this, there remains a lack of randomized controlled trials comparing the use of propofol *versus* ketamine in critically ill patients. Prior studies comparing ketamine with etomidate^(12)^ or the combination of propofol and ketamine with etomidate^(4)^ found no significant differences in clinical outcomes.

Given this uncertainty, there is a need for controlled, prospective studies to evaluate its impact on complications associated with intubation. We hypothesize that ketamine is a safe alternative for RSI in intensive care unit (ICU) patients and may be associated with a lower incidence of hypotension and other complications compared to propofol. Therefore, the primary objective of this study is to assess whether ketamine is a safer alternative to propofol for RSI by reducing the incidence of hypotension during induction in critically ill patients.

## METHODS

### Study design and participating sites

This is an investigator-initiated, randomized, open-label, pragmatic, multicenter study with two parallel groups. Critically ill patients admitted to the ICU who require orotracheal intubation will be randomly assigned to receive either ketamine or propofol as the hypnotic agent during the RSI procedure. The study design adheres to SPIRIT 2025 Statement recommendations.^(13)^

The study will be conducted at two ICUs: the *Hospital São Paulo* ICU at the *Universidade Federal de São Paulo* (UNIFESP), which has 49 beds (coordinating center), and the adult ICU of the *Hospital do Serviço Social da Indústria do Papel, Papelão e Cortiça do Estado de São Paulo* (SEPACO), with 44 beds. Both ICUs serve a mixed population of high-complexity medical and surgical patients. Both institutions have a standardized intubation protocol in place.

### Trial organization and oversight

An independent Data and Safety Monitoring Board (DSMB) will oversee the trial. The Research and Ethics Committee of UNIFESP approved this study under the number 4.846.329, followed by approval from the *Hospital SEPACO* Ethical Committee. Written informed consent will be obtained from all patients or their legally authorized representatives. The study is registered on the clinicaltrials.gov platform (identifier: NCT05092152) and is funded by a research grant from the *Conselho Nacional de Desenvolvimento Científico e Tecnológico* (CNPq).

### Eligibility criteria

Patients with a clinical indication for orotracheal intubation at the participating sites will be eligible for inclusion. Patients will be enrolled upon meeting all inclusion criteria and none of the exclusion criteria, as outlined in table 1. The study sites will maintain continuous records of all patients who meet the inclusion criteria but are not enrolled in the study, along with the reasons for their exclusion, in compliance with the Consolidated Standards of Reporting Trials (CONSORT) guidelines.

**Table 1:** Eligibility criteria

Inclusion criteria	Exclusion criteria
– Age > 18 years – Indication for orotracheal intubation for any reason	– Pregnancy – Know allergy to any of the study drugs (lidocaine, fentanyl, propofol, ketamine, or rocuronium) – History of bradycardia (heart rate < 50 bpm) or atrioventricular block without a pacemaker – Suspected intracranial hypertension – Intubation during cardiac arrest

D - Day; MAP - mean arterial pressure; MV - mechanical ventilation.

### Randomization and concealment

**Figure 1:**
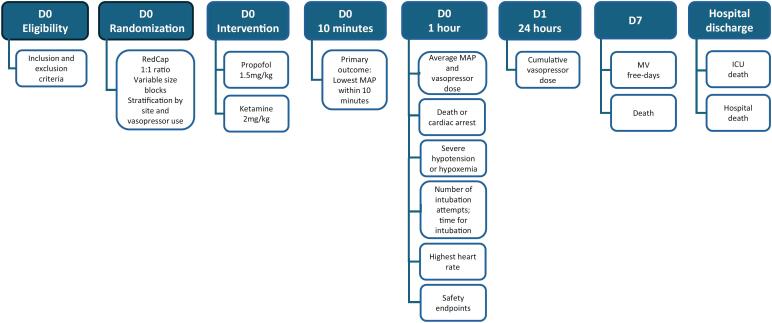
Study timeline.

Patients will be randomized to receive either ketamine or propofol via a central, web-based, automated randomization system (RedCap UNIFESP), accessible 24 hours per day. An external individual not involved in the study will generate a list using the randomization tool on random.org. Patients will be randomized in a 1:1 ratio, in blocks of variable size, stratified according to study site and use of vasopressors. The central automated system will maintain the confidentiality of the randomization sequence. Site investigators will be unaware of the block sizes, ensuring adequate concealment of the randomization sequence. The system will allow only one randomization per patient, and study team members at each site will be responsible for enrolling patients and assigning interventions. The flowchart is available in figure 1S (Supplementary Material).

### Blinding

Blinding of healthcare professionals is not feasible due to the distinct visual differences between propofol (a white lipid emulsion) and ketamine (colorless). However, data collectors, outcome assessors, and statisticians will remain blinded to group allocation to minimize bias.

### Interventions

Patients who meet the eligibility criteria will be randomized into one of two groups. Immediately following randomization, during RSI, patients in the intervention group will receive ketamine as the hypnotic agent at a dose of 2mg/kg. Patients in the control group will receive propofol at a 1.5mg/kg dose. Doses will be calculated based on the patient's weight as provided by the attending medical team.

In both groups, premedication will follow the unit's routine protocol, using either 2% lidocaine without a vasoconstrictor at a dose of 1.5mg/kg or fentanyl at 1µg/kg. All patients will receive rocuronium as a 1.2mg/kg dose of neuromuscular blocker.

The drug administration sequence will be identical in both groups. The first drug administered will be either 2% lidocaine (without vasoconstrictor) or fentanyl. After 1 minute, the induction agent (propofol or ketamine) will be administered in a bolus, followed immediately by rocuronium. The infusion rate of the drugs will not be formally specified, but sites will be oriented to follow local guidance for bolus, which will probably result in 10-second administration. Laryngoscopy will be performed 1 minute after neuromuscular blocker administration.

If the first attempt is unsuccessful, the patient will be reoxygenated before another attempt is made. If intubation remains unsuccessful after two attempts, a qualified alternate operator or adjunct devices will be utilized. If additional medications are required, the study hypnotic agent will be used, with dosing at the discretion of the attending team. All intubation steps will follow a strict protocol, and patients will undergo an intubation checklist as per routine in the participating units (Figure 2S - Supplementary Material). Continuous monitoring will be performed using electrocardiography, pulse oximetry, and invasive or non-invasive blood pressure monitoring. The monitor will proceed with measurements every 2 minutes for patients with noninvasive blood pressure measurement. The use of vasoactive drugs before induction will be initiated only if the patient's mean arterial pressure (MAP) is below 65mmHg, following the institutional protocol. Doses of vasoactive drugs can be adjusted during the procedure at the discretion of the ICU team. In both groups, general management, such as pre-oxygenation, ventilatory support, and use of adjunctive devices, will follow the ICU protocol (Supplementary Material). Data on adherence to the study protocol, including successful medication administration, will be collected for all patients who undergo intubation in both groups. Any instances patients do not receive the assigned medication will be documented, including the reason for non-administration.

### Outcomes

The study's primary outcome is the lowest MAP recorded within the first 10 minutes following the induction for intubation.

Secondary outcomes include:

– Average MAP within the first hour after induction.
– Mortality within the first hour after induction.
– Cardiac arrest within the first hour after induction.
– Occurrence of severe hypotension, defined as systolic blood pressure (SBP) < 80mmHg, within 1 hour after induction.
– Occurrence of severe hypoxemia, defined as peripheral oxygen saturation (SpO_2_) < 85%, within one hour after induction.
– Time for successful intubation.

Tertiary outcomes include:

– The number of intubation attempts.
– The highest HR within 1 hour after induction.
– The total dose of vasopressors administered within the first 24 hours, using the noradrenaline equivalent dose.
– Mean change in vasopressor dose (norepinephrine equivalent) from baseline to hour 1.
– Ventilator-free days within the first 7 days, defined as the number of days on which individuals are able to breathe spontaneously without any invasive ventilatory assistance, ascribing zero days to those who die within 7 days.
– Mortality at day 7.
– ICU mortality.
– Hospital mortality.

Safety outcomes will be assessed during the first 10 minutes following induction, and will include:

– Hypertension, defined as SBP > 180mmHg.
– Laryngospasm, as reported by the physician in charge of intubation.
– Bradycardia, defined as HR < 45 bpm.
– Arrhythmias.
– Bronchoaspiration, defined as the presence of gastric content aspirated into the airway during intubation.

All outcome data will be presented as specified on the electronic supplemental materials (Table 1S - Supplementary Material). The trial sequence is available in figure 1.

### Data collection

Demographic characteristics, comorbidities, clinical data, and severity of illness will be collected at baseline for all patients (Table 2S - Supplementary Material). Additional data on the procedure characteristics will also be collected (Table 3S - Supplementary Material). During and after the procedure, hemodynamic data will be registered at the following time points: 1 minute before induction; every 2 minutes during the first 10 minutes after the administration of the study medication; every 5 minutes up to one hour after induction (Table 4S - Supplementary Material). Doses of vasoactive drugs will also be recorded. Any adverse events occurring within the first hour post-induction, including death, cardiac arrest, arrhythmias, bronchoaspiration, and laryngospasm, will be documented.

Patients will be followed until hospital discharge, censored at day 60^th^, to determine the need for mechanical ventilation up to day 7, vital status at day 7, in the ICU, and in the hospital. Patients who are withdrawn from the study for safety reasons (excluding those who withdraw consent) will be included in the analysis on an intention-to-treat basis.

Data will be collected via electronic forms using the RedCap platform and securely stored. The investigators will complete the forms, using specific passwords for access. Training will be provided for all personnel involved in the study to ensure high-quality data collection. The principal investigator will be available to answer any questions regarding form completion. Data consistency will be periodically reviewed, and any discrepancies will be addressed by contacting the sites for clarification and correction.

### Ethical considerations

The Research and Ethics Committee of the UNIFESP approved this study under approval number 48163721.9.1001.5505, followed by approval from the *Hospital SEPACO* Ethics Committee. All patients or their legally authorized representatives will provide signed informed consent. Given the emergency nature of the study and considering that both interventions are routinely used in clinical practice, consent may be obtained retrospectively.

Patient inclusion will begin only after formal ethical approval. Investigators are committed to following all clinical best practices and adhering to the requirements of the National Health Council Resolution 510/16, ensuring the ethical conduct of the study and the safety of participants.

### Statistics

#### Sample size calculation and interim analysis

We plan to randomize an adequate number of patients to ensure 170 patients who are successfully intubated and consented to participate. Based on the assumption that some patients may be randomized but not intubated, or consent could not be obtained, we anticipate that more than 170 patients will need to be randomized. This sample size will provide 90% power with an alpha of 0.05 to detect a 10mmHg difference in the lowest MAP within the first 10 minutes after induction for intubation, assuming a standard deviation of 20mmHg. The study steering committee determined the minimum clinically important difference of 10mmHg.

An interim analysis will be performed after the inclusion of 80 patients to assess the safety profile of the intervention concerning the primary outcome. The study will not be terminated for benefit or futility unless a safety threshold of p < 0.01 is reached, particularly if significant differences in MAP favor the propofol group. This analysis will be conducted by an independent external DSMB, which will remain unblinded to group allocation. The DSMB will also review adverse events and the safety profile to provide recommendations.

#### Statistical analysis

Our primary analysis will follow a modified intention-to-treat (mITT) approach. As clinical management might change after randomization, we expect that some patients who will be randomized will not be intubated. Similarly, as we will use a deferred (opt-out) consent process, some patients (or representatives) might not consent. Thus, our mITT analysis will include only those patients who will be intubated and who consent to participate. The data analysis will be performed after submitting the protocol paper and analysis plan for publication.

Categorical variables will be expressed as counts and percentages. Continuous variables will be presented as means and standard deviations (SD) or medians with interquartile ranges (IQR, 25th -75th percentile) as appropriate. For all outcomes, including the primary outcome, we will not impute missing data, and the number of missing values will be clearly presented in tables. Treatment adherence will be reported as each group's mean (SD) number of successful medication administrations.

The primary outcome (lowest MAP within the first 10 minutes post-induction) will be analyzed using a linear regression model, adjusted for baseline MAP before induction, total vasopressor dose in the first 10 minutes, age, and a random patient intercept. A MAP value of 10mmHg (the average cardiovascular filling pressure) will be considered in patients with cardiac arrest.^(14)^ Other vasopressors will be converted to noradrenaline dose equivalents. A per-protocol analysis will be performed, including only patients who received the assigned medication. Additionally, we conducted sensitivity analysis considering the lowest measured blood pressure before cardiac arrest and an unadjusted analysis. In all primary outcome analyses, the effect estimate will be expressed by the mean difference with 95% confidence intervals (95%CI) and p values reported.

For secondary binary and exploratory outcomes, logistic regression will be used, with the effect estimate expressed as odds ratios (ORs) and 95%CIs, with their respective p values. Deaths within the specified ranges will be considered zero free days for outcomes related to free days (ventilator-free days in 7 days). Logistic regression models for ordinal data will report proportional ORs. The frequency of adverse events will be expressed as counts and percentages, and comparisons will be made using the Chi-squared test for categorical variables. Secondary outcomes will not be adjusted for covariates.

For the primary outcome, a pre-specified subgroup analysis will evaluate the following groups: patients < 70 *versus* ≥ 70 years old; patients with a Sequential Organ Failure Assessment (SOFA) score < 8 *versus* ≥ 8 points; patients on vasopressor *versus* without vasopressors; and patients with acute respiratory distress syndrome (ARDS) *versus* those without ARDS. Each subgroup analysis will be performed using linear regression, adjusted for baseline MAP before induction, total vasopressor dose in the first 10 minutes, age, and a random patient intercept. Effect estimates will be reported as mean differences with 95%CIs for each subgroup, with respective interaction p values. We will not adjust for multiplicity.

We will consider a significant two-tailed p level ≤ 0.05. Analysis will be performed using R software in the available version (R Foundation for Statistical Computing), Vienna, Austria.

### Safety and adverse events

Given the severely ill ICU population undergoing intubation, we anticipate potential complications. These events will only be considered adverse if they are potentially related to the study treatment or deemed unexpected by the attending physician and unrelated to the underlying critical illness that led to intubation. (Table 5S - Supplementary Material)

Serious adverse events will be those that are life-threatening, result in death, lead to prolonged hospitalization, or cause severe and permanent disability. They will be adjudicated by physicians independent from the study team and reported to the DSMB and ethics committees.

### Confidentiality

Only initials and hospital registration numbers will be recorded. All clinical and study-related information will be treated confidentially. Clinical records will be stored in areas with restricted access, limited to investigators and authorized data collection staff.

### Role of sponsor source and conflicts of interest

This trial is investigator-led, with ketamine and propofol provided by Cristalia (São Paulo, Brazil). Flávia Ribeiro Machado has a research grant from the *Conselho Nacional de Desenvolvimento Científico e Tecnológico* (CNPq). Neither Cristalia nor CNPq is involved in the study design, data analysis, manuscript preparation, or decision to submit results for publication. The authors declare no conflicts of interest.

### Data dissemination policies

The study results will be disseminated through scientific communications, including presentations at medical congresses and publication in peer-reviewed journals. All principal investigators involved at the centers will be listed as authors, provided they meet international authorship criteria.

## DISCUSSION

This study protocol outlines a randomized, open-label, pragmatic, bicentric trial designed to evaluate the effects of ketamine versus propofol as the hypnotic agent in critically ill patients undergoing RSI. The primary outcome of the study is the lowest MAP within 10 minutes after induction for intubation. Secondary outcomes include various cardiovascular and respiratory parameters, adverse events, and patient-related outcomes such as ventilator-free days and mortality.

The findings from this study could provide important insights into the comparative efficacy and safety of two commonly used anesthetic agents in a high-risk ICU setting and have the potential to significantly influence clinical practice. If ketamine is shown to be superior to propofol in maintaining hemodynamic stability without compromising sedation quality, it may become the preferred choice for intubation in this patient population. On the other hand, if propofol is found to be equally effective but with fewer adverse events or a more favorable safety profile, it could continue to be a viable option in certain circumstances.

Our study design has several strengths. The study will be conducted in two ICUs serving a diverse population of high-complexity medical and surgical patients. Additionally, using randomization with concealment of allocation through a centralized, web-based system helps minimize selection bias. Our predefined primary and secondary outcomes are clinically relevant and directly tied to patient safety and treatment efficacy. Furthermore, the protocol incorporates a well-defined safety monitoring framework, with an independent DSMB overseeing the study's progress and ensuring the safety of participants.

There are also several limitations. The open-label design may introduce performance bias. Although blinding is not possible for the healthcare providers administering the medications, efforts to blind outcome assessors, data collectors, and statisticians will help minimize bias in outcome assessment.

## CONCLUSIONS

The PROMINE study will provide more evidence regarding the optimal choice of hypnotic drug for rapid sequence intubation in critically ill patients, enhancing current knowledge about the effects, particularly hemodynamics, which may arise from propofol and ketamine in this context.

## Supplementary Materials

Supplementary material 1

## References

[1] Acquisto NM, Mosier JM, Bittner EA, Patanwala AE, Hirsch KG, Hargwood P (2023). Society of Critical Care Medicine Clinical Practice Guidelines for Rapid Sequence Intubation in the Critically Ill Adult Patient. Crit Care Med.

[2] Gholipour Baradari A, Firouzian A, Zamani Kiasari A, Aarabi M, Emadi SA, Davanlou A (2016). Effect of etomidate versus combination of propofol-ketamine and thiopental-ketamine on hemodynamic response to laryngoscopy and intubation: a randomized double blind clinical trial. Anesth Pain Med.

[3] Russotto V, Myatra SN, Laffey JG, Tassistro E, Antolini L, Bauer P (2021). INTUBE Study Investigators. Intubation practices and adverse peri-intubation events in critically ill patients from 29 countries. JAMA.

[4] Smischney NJ, Nicholson WT, Brown DR, Gallo De Moraes A, Hoskote SS, Pickering B (2019). Ketamine/propofol admixture vs etomidate for intubation in the critically ill: KEEP PACE randomized clinical trial. J Trauma Acute Care Surg.

[5] Sereeyotin J, Yarnell C, Mehta S (2025). Sedation practices in patients intubated in the emergency department compared with those in patients in the intensive care unit. Crit Care Sci.

[6] Midega TD, Chaves RC, Ashihara C, Alencar RM, Queiroz VN, Zelezoglo GR (2022). Ketamine use in critically ill patients: a narrative review. Rev Bras Ter Intensiva.

[7] Mohr NM, Pape SG, Runde D, Kaji AH, Walls RM, Brown CA (2020). Etomidate use is associated with less hypotension than ketamine for emergency department sepsis intubations: a NEAR cohort study. Acad Emerg Med.

[8] Green RS, Turgeon AF, McIntyre LA, Fox-Robichaud AE, Fergusson DA, Doucette S (2015). Canadian Critical Care Trials Group (CCCTG). Postintubation hypotension in intensive care unit patients: A multicenter cohort study. J Crit Care.

[9] Singh A, Iyer KV, Maitra S, Khanna P, Sarkar S, Ahuja V (2022). Ketamine and dexmedetomidine (Keto-dex) or ketamine and propofol (Keto-fol) for procedural sedation during endoscopic retrograde cholangiopancreatography: which is safer? A randomized clinical trial. Indian J Gastroenterol.

[10] Ferguson I, Buttfield A, Burns B, Reid C, Shepherd S, Milligan J, Australasian College for Emergency Medicine Clinical Trials Network (2022). Fentanyl versus placebo with ketamine and rocuronium for patients undergoing rapid sequence intubation in the emergency department: the FAKT study-A randomized clinical trial. Acad Emerg Med.

[11] Ishimaru T, Goto T, Takahashi J, Okamoto H, Hagiwara Y, Watase H, Japanese Emergency Medicine Network Investigators (2019). Association of ketamine use with lower risks of post-intubation hypotension in hemodynamically-unstable patients in the emergency department. Sci Rep.

[12] Jabre P, Combes X, Lapostolle F, Dhaouadi M, Ricard-Hibon A, Vivien B, KETASED Collaborative Study Group (2009). Etomidate versus ketamine for rapid sequence intubation in acutely ill patients: a multicentre randomised controlled trial. Lancet.

[13] Chan AW, Boutron I, Hopewell S, Moher D, Schulz KF, Collins GS (2025). SPIRIT 2025 statement: updated guideline for protocols of randomised trials. BMJ.

[14] Rothe CF (1993). Mean circulatory filling pressure: its meaning and measurement. J Appl Physiol (1985).

