# Supplementary material for: Prospective, randomized, controlled trial comparing PROpofol versus KetaMINE in rapid sequence intubation in critically ill patients (PROMINE): protocol paper and statistical analysis plan
Source: Crit Care Sci. 2025 Nov 3;37:e20250133. doi: 10.62675/2965-2774.20250133 (PMC12614951; doi:10.62675/2965-2774.20250133)
Supplement: Supplementary material 1 [file 2965-2774-ccsci-37-e20250133-suppl01.pdf]

Critical Care Science
https://doi.org/10.62675/2965-2774.20250133

SUPPLEMENTARY MATERIAL

Prospective, randomized, controlled trial comparing PROpofol versus KetaMINE in rapid sequence intubation in critically ill patients (PROMINE): protocol paper and statistical analysis plan

Raysa Cristina Schmidt[1,2], Fernando Godinho Zampieri[1], Fernando Jose da Silva Ramos[1,2], Felipe Santos Cavatoni Serra[1], Lucas Petri Damiani[1], Flávio Geraldo Rezende de Freitas[1,2,3], Flávia Ribeiro Machado[1,2], for the PROMINE Investigators

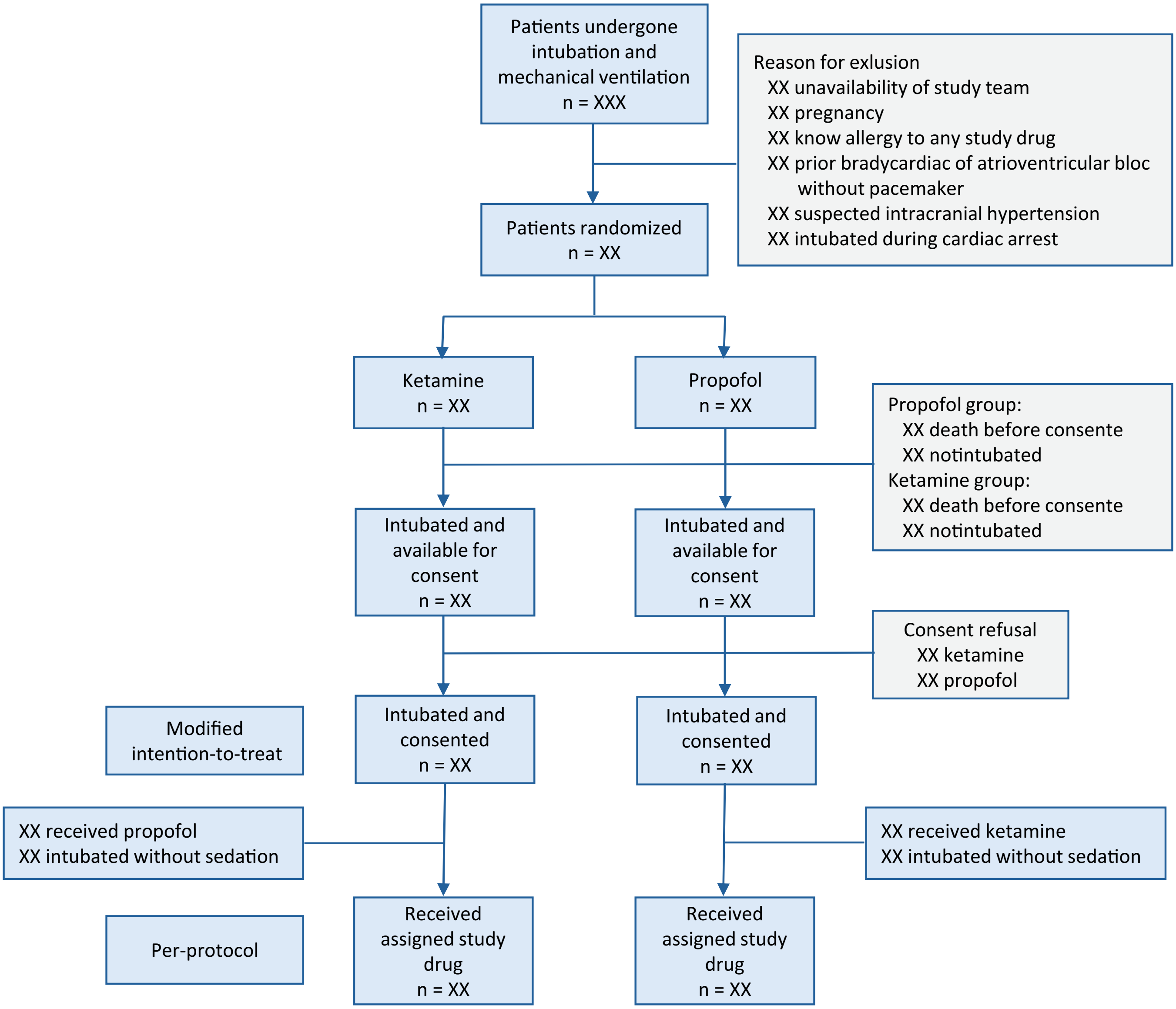

Figure 1S - Study flowchart.

This is an open access article under the CC BY license (https://creativecommons.org/licenses/by/4.0/).

Initial patient assessment
- ▢ Patient can be included in the PROMINA study
- ▢ Assessment of difficult airway predictors
- ▢ Availability of a difficult airway cart/trolley
- ▢ Assessment of hemodynamic status
- ▢ Ensure adequate pre-oxygenation
- ▢ Removal of dentures

Equipment check
- ▢ Laryngoscope with working 3 and 4 curved blades
- ▢ Determine if a video laryngoscope is necessary
- ▢ Predicted orotracheal tube tested numbers 7.5; 8.0 or 8.5
- ▢ Optional tubes available: numbers 7.5; 8.0 or 8.5
- ▢ Syringe for cuff inflation
- ▢ Material to fix the orotracheal tube
- ▢ Endotracheal tube stylet readily available
- ▢ Bag-valve mask (BVM) connected to supplemental oxygen
- ▢ Oropharyngeal airways (Guedel airways) sizes 3 and 4 readily available
- ▢ Suction equipment readily available and functional
- ▢ Mechanical ventilator prepared and ready for use

Monitoring
- ▢ Medications for endotracheal intubation drawn up and ready
- ▢ Ensure proper patient positioning
- ▢ Functional cardiac monitor (ECG)
- ▢ Functional pulse oximeter, easily accessible and placed on a limb without a blood pressure cuff
- ▢ Functional blood pressure monitoring: cuff or arterial line (MAP)
- ▢ Functional intravenous access
- ▢ Monitor visible to the physician performing the procedure or an assisting physician

Proper patient positioning
- ▢ Supine position
- ▢ Suboccipital support (pillow or pad), sniff position

During the procedure
- ▢ Monitor vital signs every 2 minutes for the first 10 minutes post-intubation
- ▢ Monitor vital signs every 5 minutes between 10 and 60 minutes post-intubation

After the procedure
- ▢ Ensure secure fixation of the endotracheal tube
- ▢ Confirm endotracheal tube placement: capnography and auscultation
- ▢ Elevate the head of the bed to 30 degrees
- ▢ Assess endotracheal tube cuff pressure
- ▢ Evaluate hemodynamic parameters after completion of endotracheal intubation
- ▢ Order a post-intubation chest X-ray and arterial blood gas analysis
- ▢ Assess the need for continuous sedation

Figure 2S - Intubation checklist

GENERAL MANAGEMENT DURING INTUBATION

The intubation procedure will follow intensive care unit (ICU) protocol for both groups. Patients will undergo pre-oxygenation with 100% oxygen via non-invasive ventilation, high-flow nasal cannula, or a non-rebreathing mask, depending on the available support at the time of intubation, at the discretion of the attending physician. If necessary, bag-valve-mask ventilation may be employed at any point during the procedure. If the first attempt at intubation is unsuccessful, re-oxygenation will occur, and a second attempt will be made. If intubation is not achieved after two attempts, the procedure will be continued by another qualified professional or with the use of adjunctive devices. A stethoscope will be available for confirming intubation, and capnography and thoracic ultrasonography will be utilized if available. Patients' management will be left to the discretion of the attending physicians

ADVERSE EVENTS

According to the medication leaflets, known adverse events related to ketamine include hypertension or hypotension, tachycardia or bradycardia, *delirium*, confusion, tonic-clonic movements, nausea and vomiting, arrhythmia, laryngospasm, diplopia, nystagmus, increased intraocular pressure, rash, sialorrhea, and morbilliform eruption. Known adverse events related to propofol include pain at the infusion site, phlebitis, bradycardia, hypotension, nausea and vomiting, epileptiform movements including seizures and opisthotonos, acute hypersensitivity reactions, and possible edema and bronchospasm. Bronchoaspiration, defined as the presence of gastric contents in the airway or vomiting during or up to 10 minutes after induction will be recorded.

Table 1S - Primary, secondary and tertiary outcomes – overall treatment effect

Outcomes	Ketamine (n = xx)	Propofol (n = xx)	Effect measure		p value
			Type	95%CI	
Primary outcome median (IQR)					
Lowest MAP within 10 minutes*, median (IQR)	xx.x ± xx.x	xx.x ± xx.x	MD†	x.x [x.x - x.x]	x.xx
Secondary outcome					
Average MAP within 1st hour, median (IQR)	xx.x ± xx.x	xx.x ± xx.x	MD	x.x [x.x - x.x]	x.xx
Mortality within 1st hour, n (%)	xx (xx)	xx (xx)	OR	x.x [x.x - x.x]	x.xx
Cardiac arrest within 1st hour, n (%)	xx (xx)	xx (xx)	OR	x.x [x.x to x.x]	x.xx
Severe hypotension, n (%)	xx (xx)	xx (xx)	OR	x.x [x.x - x.x]	x.xx
Severe hypoxemia, n (%)	xx (xx)	xx (xx)	OR	x.x [x.x - x.x]	x.xx
Time for successful intubation, median (IQR)	XXXXX	XXXXX	MD	x.x [x.x - x.x]	x.xx
Tertiary outcomes					
Number of intubation attempts, median (IQR)			MD	x.x [x.x - x.x]	x.xx
1	xx (xx)	xx (xx)			
2	xx (xx)	xx (xx)			
3	xx (xx)	xx (xx)			
4 or more	xx (xx)	xx (xx)			
Highest heart rate within 1 hour, mean ± SD	xx.x ± xx.x	xx.x ± xx.x	MD	x.x [x.x - x.x]	x.xx
Total dose of vasopressors (within 24 hours)			MD	x.x [x.x - x.x]	x.xx
Mean change in vasopressor dose from baseline to hour 1			MD	x.x [x.x - x.x]	x.xx
Ventilator-free days in 7 days, mean ± SD	xx.x ± xx.x	xx.x ± xx.x	POR	x.x [x.x - x.x]	x.xx
7-day mortality, n (%)	xx (xx)	xx (xx)	OR	x.x [x.x - x.x]	x.xx
ICU mortality, n (%)	xx (xx)	xx (xx)	OR	x.x [x.x - x.x]	x.xx

Continue...

...continuation

Hospital mortality	xx (xx)	xx (xx)	OR	x.x [x.x - x.x]	x.xx
Safety outcomes, n (%)					
Hypertension					
Laryngospasm					
Bradycardia					
Arrhythmias					
Bronchoaspiration					

95%CI - 95% confidence interval; MAP - mean arterial pressure; MD - mean difference; OR - odds ratio; POR - proportional odds ratio; ICU - intensive care unit. *10mmHg was considered in the primary outcome of patients with cardiac arrest within 10minutes. † Linear model adjusted for baseline mean arterial pressure, total vasopressor dose in the first 10 minutes vasopressor dose, age, and a random patient intercept.

Table 2S - Baseline characteristics

Variables	Ketamine n = XX	Propofol n = XX
Age, years, median (IQR)	XXXXX	XXXXX
Sex (female), n (%)	XXXXX	XXXXX
Weight (Kg), mean (SD)	XXXXX	XXXXX
Time from ICU admission to randomization, median (IQR)	XXXXX	XXXXX
SAPS 3, median (IQR)	XXXXX	XXXXX
SOFA D0, median (IQR)	XXXXX	XXXXX
Type of admission		
Clinical, n (%)	XXXXX	XXXXX
Elective surgery, n (%)	XXXXX	XXXXX
Emergency surgery, n (%)	XXXXX	XXXXX
Previous admisson		
Ward, n (%)	XXXXX	XXXXX
Emergency department, n (%)	XXXXX	XXXXX
Operating room, n (%)	XXXXX	XXXXX
Comorbidities, n (%)		
Diabetes mellitus, n (%)	XXXXX	XXXXX
Heart failure, n (%)	XXXXX	XXXXX
Chronic obstructive pulmonary disease, n (%)	XXXXX	XXXXX
Solid non-metastatic cancer, n (%)	XXXXX	XXXXX
Solid metastatic cancer, n (%)	XXXXX	XXXXX
Haematological malignancy, n (%)	XXXXX	XXXXX
Chronic kidney disease, n (%)	XXXXX	XXXXX
Cirrhosis, n (%)	XXXXX	XXXXX
HIV/AIDS, n (%)	XXXXX	XXXXX
Immunosuppression, n (%)	XXXXX	XXXXX
Charlson Comorbidity Index, median (IQR)	XXXXX	XXXXX

IQR interquartile range; ICU - intensive care unit; SD standard deviation; SAPS 3 - Simplified Acute Physiology Score 3; SOFA - Sequential Organ Failure Assessment; HIV/AIDS - human immunodeficiency virus/acquired immunodeficiency syndrome.

Table 3S - Procedure characteristics

Variables	Ketamine<br>n = XX	Propofol<br>n = XX
Primary indication for intubation		
Respiratory failure, n (%)	XXXXX	XXXXX
Reduced level of conciousness, n (%)	XXXXX	XXXXX
Shock, n (%)	XXXXX	XXXXX
Elective procedure, n (%)	XXXXX	XXXXX
Airway protection, n (%)	XXXXX	XXXXX
Other, n (%)	XXXXX	XXXXX
Cormack classification		
1	XXXXX	XXXXX
II	XXXXX	XXXXX
III	XXXXX	XXXXX
IV	XXXXX	XXXXX
Not reported	XXXXX	XXXXX
Vital signs immediately before induction		
Systolic arterial pressure (mmHg), mean (SD)	XXXXX	XXXXX
Mean arterial pressure (mmHg), mean (SD)	XXXXX	XXXXX
Heart rate (bpm), mean (SD)	XXXXX	XXXXX
Peripheral oxygen saturation (%), mean (SD)	XXXXX	XXXXX
Glasgow coma score, median (IQR)	XXXXX	XXXXX
Pre oxygenation support		
Room air	XXXXX	XXXXX
Nasal canula	XXXXX	XXXXX
Facial mask	XXXXX	XXXXX
High flow	XXXXX	XXXXX
Non invasive ventilation/HFNC	XXXXX	XXXXX
Use of vasopressors (immediately before intubation)		
Norepinephrine, n (%)	XXXXX	XXXXX
Dose (mcg/Kg/min), mean (SD)	XXXXX	XXXXX
Epinephrine, n (%)	XXXXX	XXXXX
Dose (mcg/Kg/min), mean (SD)	XXXXX	XXXXX
Vasopressine, n (%)	XXXXX	XXXXX
Dose (UI/min), mean (SD)	XXXXX	XXXXX
Dobutamine, n (%)	XXXXX	XXXXX
Dose (mcg/Kg/min), mean (SD)	XXXXX	XXXXX
None, n (%)	XXXXX	XXXXX
Medications used in induction		
Pre-medication, dose (mcg/Kg, UI, mg/Kg), n (%)		

Continue...

...continuation

Fentanyl, n (%)	XXXXX	XXXXX
Dose (mcg/Kg), mean (SD)	XXXXX	XXXXX
Lidocaine, n (%)	XXXXX	XXXXX
Dose (mg/Kg), mean (SD)	XXXXX	XXXXX
None, n (%)	XXXXX	XXXXX
Neuromuscular blocker, dose (mcg/Kg), n (%)		
Rocuronium, n (%)	XXXXX	XXXXX
Dose (mg/Kg), mean (SD)	XXXXX	XXXXX
Succinylcoline, n (%)	XXXXX	XXXXX
Dose (mg/Kg), mean (SD)	XXXXX	XXXXX
None, n (%)	XXXXX	XXXXX
Hypnotic drugs, dose (mcg/Kg), n (%)		
Ketamine, n (%)	XXXXX	XXXXX
Dose (mg/Kg), mean (SD)	XXXXX	XXXXX
Propofol, n (%)	XXXXX	XXXXX
Dose (mg/Kg), mean (SD)	XXXXX	XXXXX
Level of training (first attempt)		
Resident physician, n (%)	XXXXX	XXXXX
Uncertified physician, n (%)	XXXXX	XXXXX
Board certified intensivist, n (%)	XXXXX	XXXXX

IQR -interquartile range; SD - standard deviation; HFNC - high-flow nasal cannula.

Table 4S - Hemodynamic data during and one hour after the procedure

Variable	Time point	Ketamina	Propofol	Dif. Ketamina - Propofol [95%CI]
MAP	-1	xx.x (xx.x), n = xx	xx.x (xx.x), n = xx	x.x [x.x to x.x]
	0	xx.x (xx.x), n = xx	xx.x (xx.x), n = xx	x.x [x.x to x.x]
	2	xx.x (xx.x), n = xx	xx.x (xx.x), n = xx	x.x [x.x to x.x]
	4	xx.x (xx.x), n = xx	xx.x (xx.x), n = xx	x.x [x.x to x.x]
	6	xx.x (xx.x), n = xx	xx.x (xx.x), n = xx	x.x [x.x to x.x]
	8	xx.x (xx.x), n = xx	xx.x (xx.x), n = xx	x.x [x.x to x.x]
	10	xx.x (xx.x), n = xx	xx.x (xx.x), n = xx	x.x [x.x to x.x]
	15	xx.x (xx.x), n = xx	xx.x (xx.x), n = xx	x.x [x.x to x.x]
	20	xx.x (xx.x), n = xx	xx.x (xx.x), n = xx	x.x [x.x to x.x]
	25	xx.x (xx.x), n = xx	xx.x (xx.x), n = xx	x.x [x.x to x.x]
	30	xx.x (xx.x), n = xx	xx.x (xx.x), n = xx	x.x [x.x to x.x]
	35	xx.x (xx.x), n = xx	xx.x (xx.x), n = xx	x.x [x.x to x.x]
	40	xx.x (xx.x), n = xx	xx.x (xx.x), n = xx	x.x [x.x to x.x]
	45	xx.x (xx.x), n = xx	xx.x (xx.x), n = xx	x.x [x.x to x.x]
	50	xx.x (xx.x), n = xx	xx.x (xx.x), n = xx	x.x [x.x to x.x]
	55	xx.x (xx.x), n = xx	xx.x (xx.x), n = xx	x.x [x.x to x.x]
	60	xx.x (xx.x), n = xx	xx.x (xx.x), n = xx	x.x [x.x to x.x]
SBP	-1	xx.x (xx.x), n = xx	xx.x (xx.x), n = xx	x.x [x.x to x.x]
	0	xx.x (xx.x), n = xx	xx.x (xx.x), n = xx	x.x [x.x to x.x]
	2	xx.x (xx.x), n = xx	xx.x (xx.x), n = xx	x.x [x.x to x.x]
	4	xx.x (xx.x), n = xx	xx.x (xx.x), n = xx	x.x [x.x to x.x]
	6	xx.x (xx.x), n = xx	xx.x (xx.x), n = xx	x.x [x.x to x.x]
	8	xx.x (xx.x), n = xx	xx.x (xx.x), n = xx	x.x [x.x to x.x]
	10	xx.x (xx.x), n = xx	xx.x (xx.x), n = xx	x.x [x.x to x.x]
	15	xx.x (xx.x), n = xx	xx.x (xx.x), n = xx	x.x [x.x to x.x]
	20	xx.x (xx.x), n = xx	xx.x (xx.x), n = xx	x.x [x.x to x.x]
	25	xx.x (xx.x), n = xx	xx.x (xx.x), n = xx	x.x [x.x to x.x]
	30	xx.x (xx.x), n = xx	xx.x (xx.x), n = xx	x.x [x.x to x.x]
	35	xx.x (xx.x), n = xx	xx.x (xx.x), n = xx	x.x [x.x to x.x]
	40	xx.x (xx.x), n = xx	xx.x (xx.x), n = xx	x.x [x.x to x.x]
	45	xx.x (xx.x), n = xx	xx.x (xx.x), n = xx	x.x [x.x to x.x]
	50	xx.x (xx.x), n = xx	xx.x (xx.x), n = xx	x.x [x.x to x.x]
	55	xx.x (xx.x), n = xx	xx.x (xx.x), n = xx	x.x [x.x to x.x]
	60	xx.x (xx.x), n = xx	xx.x (xx.x), n = xx	x.x [x.x to x.x]
HR	-1	xx.x (xx.x), n = xx	xx.x (xx.x), n = xx	x.x [x.x to x.x]
	0	xx.x (xx.x), n = xx	xx.x (xx.x), n = xx	x.x [x.x to x.x]
	2	xx.x (xx.x), n = xx	xx.x (xx.x), n = xx	x.x [x.x to x.x]
	4	xx.x (xx.x), n = xx	xx.x (xx.x), n = xx	x.x [x.x to x.x]
	6	xx.x (xx.x), n = xx	xx.x (xx.x), n = xx	x.x [x.x to x.x]
	8	xx.x (xx.x), n = xx	xx.x (xx.x), n = xx	x.x [x.x to x.x]
	10	xx.x (xx.x), n = xx	xx.x (xx.x), n = xx	x.x [x.x to x.x]
	15	xx.x (xx.x), n = xx	xx.x (xx.x), n = xx	x.x [x.x to x.x]

Continue...

...continuation

	20	xx.x (xx.x), n = xx	xx.x (xx.x), n = xx	x.x [x.x to x.x]
	25	xx.x (xx.x), n = xx	xx.x (xx.x), n = xx	x.x [x.x to x.x]
	30	xx.x (xx.x), n = xx	xx.x (xx.x), n = xx	x.x [x.x to x.x]
	35	xx.x (xx.x), n = xx	xx.x (xx.x), n = xx	x.x [x.x to x.x]
	40	xx.x (xx.x), n = xx	xx.x (xx.x), n = xx	x.x [x.x to x.x]
	45	xx.x (xx.x), n = xx	xx.x (xx.x), n = xx	x.x [x.x to x.x]
	50	xx.x (xx.x), n = xx	xx.x (xx.x), n = xx	x.x [x.x to x.x]
	55	xx.x (xx.x), n = xx	xx.x (xx.x), n = xx	x.x [x.x to x.x]
	60	xx.x (xx.x), n = xx	xx.x (xx.x), n = xx	x.x [x.x to x.x]
SaO_2	-1	xx.x (xx.x), n = xx	xx.x (xx.x), n = xx	x.x [x.x to x.x]
	0	xx.x (xx.x), n = xx	xx.x (xx.x), n = xx	x.x [x.x to x.x]
	2	xx.x (xx.x), n = xx	xx.x (xx.x), n = xx	x.x [x.x to x.x]
	4	xx.x (xx.x), n = xx	xx.x (xx.x), n = xx	x.x [x.x to x.x]
	6	xx.x (xx.x), n = xx	xx.x (xx.x), n = xx	x.x [x.x to x.x]
	8	xx.x (xx.x), n = xx	xx.x (xx.x), n = xx	x.x [x.x to x.x]
	10	xx.x (xx.x), n = xx	xx.x (xx.x), n = xx	x.x [x.x to x.x]
	15	xx.x (xx.x), n = xx	xx.x (xx.x), n = xx	x.x [x.x to x.x]
	20	xx.x (xx.x), n = xx	xx.x (xx.x), n = xx	x.x [x.x to x.x]
	25	xx.x (xx.x), n = xx	xx.x (xx.x), n = xx	x.x [x.x to x.x]
	30	xx.x (xx.x), n = xx	xx.x (xx.x), n = xx	x.x [x.x to x.x]
	35	xx.x (xx.x), n = xx	xx.x (xx.x), n = xx	x.x [x.x to x.x]
	40	xx.x (xx.x), n = xx	xx.x (xx.x), n = xx	x.x [x.x to x.x]
	45	xx.x (xx.x), n = xx	xx.x (xx.x), n = xx	x.x [x.x to x.x]
	50	xx.x (xx.x), n = xx	xx.x (xx.x), n = xx	x.x [x.x to x.x]
	55	xx.x (xx.x), n = xx	xx.x (xx.x), n = xx	x.x [x.x to x.x]
	60	xx.x (xx.x), n = xx	xx.x (xx.x), n = xx	x.x [x.x to x.x]

MAP - mean arterial pressure; SBP - systolic blood pressure; HR - heart rate; SaO_2 – oxygen saturation.

Table 5S - Adverse events according to the group

Variable	Ketamine<br>n = XX	Propofol<br>n = XX	Total<br>n = XX	p value
Adverse events, n (%)	xx/xx (xx,x%)	xx/xx (xx,x%)	xx/xx (xx,x%)	x.xxx
Serious adverse events, n (%)	xx/xx (xx,x%)	xx/xx (xx,x%)	xx/xx (xx,x%)	x.xxx
XXXXXX	xx/xx (xx,x%)	xx/xx (xx,x%)	xx/xx (xx,x%)	x.xxx
XXXXXX	xx/xx (xx,x%)	xx/xx (xx,x%)	xx/xx (xx,x%)	x.xxx
XXXXXX	xx/xx (xx,x%)	xx/xx (xx,x%)	xx/xx (xx,x%)	x.xxx
